# Community leaders’ perceptions of and responses to intimate partner violence in Northwestern Ghana

**DOI:** 10.1371/journal.pone.0262870

**Published:** 2022-03-01

**Authors:** Isaac Dery, Constance A. Akurugu, Cuthbert Baataar

**Affiliations:** 1 Department of African and General Studies, Simon Diedong Dombo University of Business and Integrated Development Studies, Wa, Ghana; 2 Department of Community Development, Faculty of Planning and Land Management, Simon Diedong Dombo University of Business and Integrated Development Studies, Wa, Ghana; Washington University in St. Louis, UNITED STATES

## Abstract

Calls to engage community leaders in preventing gender-based violence against women have gained global prominence in recent years. Situated within the growing calls for greater community leaders’ engagement, this article problematizes the assumptions that efforts to mobilize community gatekeepers in violence prevention are likely to yield better results. Drawing inspiration from decolonial African feminist perspectives coupled with five focus group discussions conducted with 30 community leaders in the patriarchal setting of Northwestern Ghana, this article highlights the potential limitations of these assumptions by paying attention to the multiple ways; albeit subtly, in which community leaders as cultural gatekeepers may individually or collectively reproduce and sustain dominant cultural tropes that normalize violence against women. Our findings show that cultural gatekeepers’ perspectives on and their approaches to addressing violence against women risk normalizing and perpetuating it. If policy makers, development practitioners, and researchers are to adequately address the violence of men, a useful starting point is to build on community leaders’ perspectives, attitudes, and responses to violence as a collective issue. By building on these, we will be able to challenge and deconstruct the multiple ways in which community leaders’ approaches to addressing violence are reinforcing gendered subordination.

## Introduction

Globally, there is no doubt that long-standing feminist theorizing, activism, and scholarship have provided an important intellectual and political impetus through which gendered subjectivities, including gender-based violence have been brought to the forefront of public debate. Feminism(s) as an intellectual and political enterprise has created useful possibilities through which the everyday gendered subjectivities, experiences, and struggles of women may be interpreted [1]. As feminist discourses and theorizing continue to offer authoritative appeal to diverse disciplinary circles, it has become even more urgent than ever before to scrutinize and challenge deep-seated structures of patriarchy and intersecting inequalities that continue to disadvantage women across the world [2]. The call for greater understanding of women’s everyday experiences and how they navigate gendered subjectivities is not entirely new; it has been a central focus of feminists’ politicking and advocacy since the late 1960s and early 1970s. Since the late 1960s and early 1970s, there has been renewed politicization among feminists, especially second-wave feminists on the need to dismantle boundaries between the private and public spaces through which gendered subjectivities are reproduced and made sense of on a daily basis.

Through the proliferation of feminist writings and activism in both resource-poor and more industrialized Northern countries, there is now a vibrant body of ideas, knowledge, and scholarship that foregrounds women’s everyday gendered experiences, especially on gender-based violence as part of the broader socio-political and cultural contexts in which women’s lives are embedded. While feminist scholarship and activism have engineered stronger waves of global commitment to address violence perpetrated against women, it is evident that about 35% of women across the globe have ever experienced different forms of violence [3].

In view of the devastating consequences of violence against women, especially intimate partner violence (IPV), it has become increasingly important that intersecting factors that normalize violence, including patriarchal structures, ideologies, gendered practices, and socio-cultural norms should be deconstructed and transformed. Patriarchal ideologies, gendered practices, and socio-cultural norms are discursive scripts that are taken to define what is acceptable behavior for men and women, boys and girls within a specific geographical and cultural context. These discursive constructs are often used to sustain power hierarchies and structures of inequalities between and among diverse social categories [4, 5]. The power hierarchies and imbalances play crucial roles in shaping the dynamics and patterns of violence, including who is at the risk of experiencing violence and who is culpable of perpetrating violence [6, 7]. In the process, the question on why and how violence is gendered becomes critical to feminist theorizing and knowledge production [8, 9]. These authors have argued that social conditions and structures of patriarchy create durable patterns of victimization in which bodies mapped as ‘masculine’ or ‘feminine’ are central to the very configuration and meaning-making of violence. Building on such insights, the aim of this article is to critically foreground culturally nuanced understanding of IPV from community leaders. We focus on understanding the perspectives of community leaders as they serve as important stakeholders in processes and interventions aimed at addressing violence broadly at the local level.

In male dominated societies characterized by entrenched patriarchal norms and systemic inequalities, the phenomenon of gendered violence is a difficult issue to address. This is particularly true in northwestern Ghana, where a close-knit family structure is valued and violence against women, especially IPV is considered as a ‘private’ or family issue and mostly normalized. In these patriarchal and patrilineal societies, understanding how different actors may perceive IPV is important for a deeper appreciation of the contextual complexities of violence. Therefore, this article addresses the following questions: How do cultural gatekeepers, such as local chiefs, religious leaders, traditional rulers, and other opinion leaders make sense of the perpetration of violence? In a context with limited support systems for victims, what roles are cultural gatekeepers playing in addressing the violence of men? How are these gatekeepers contributing to challenge or reinforce patriarchal norms and heteronormative arrangements that encourage violence against women to flourish?

## Masculinities and intimate partner violence

The literature on the nexus between constructions of masculinities and intimate partner violence is so vast that we are unable to offer a comprehensive review within the limited space of this paper. What we do aim to achieve is to foreground an understanding of why certain versions of masculinity are valorized as acts of violence against women in general and against other men as a productive embodiment of masculinity. Across the world, and in particular, in gender inequitable societies, there is evidence to suggest that discursive cultural scripts and ideologies pertaining to local constructions of masculinity may contribute to the normalization and acceptance of IPV [4, 5, 10–13]. Such evidence foregrounds how problematic notions of masculinity have long been associated with men’s perpetration of and/or silence on multiple forms of violent behavior. Acts of violence may be used by men to enforce women’s compliance with dominant cultural norms and scripts that police notions of credible femininity. In a context where culturally constructed notions of hegemonic masculinity; of being a breadwinner and family provider remain deeply aspirational for most men as a result of limited opportunities [14], IPV may become a recuperative apparatus to re-establish deflated masculine identity [8, 10, 15]. In societies in which women experience widespread economic inequalities and intractable poverty, violence can be a really complicated phenomenon to navigate, and the prospects for such women to leave a violent relationship is likely to be highly compromised. Against the backdrop of such complexities, we would suggest that a more nuanced theorizing that takes seriously the multifaceted experiences of women, and how such experiences are located within a broader system of power relationships and struggles is inevitable.

Gendered socialization processes become important means through which men may perceive a sense of cultural entitlement to control women in order to maintain the patriarchal order. Violence or threats of violence may be pursued as an active resource to ensure that the cultural hegemony of men is maintained and that the position of women is defined by subservience and submissiveness. Different socialization agents and institutions, including churches, mosques, communities, families, and government departments may become complicit in perpetuating a culture of violence and impunity. Scholars such as [15–17] have maintained that gendered norms, including culturally constructed notions of masculinity and femininity do not only organize and shape people’s interpersonal interactions and relationships, but that institutions may draw on these socially constructed registers to solidify and exacerbate existing unequal practices and power hierarchies. This may lead to the institutionalization of impunity in which violence becomes normalized and impunity glorified. In the context of Ghana, for example, various religious leaders (e.g., Pastors, Imams) and cultural gatekeepers (e.g., chiefs, traditional rulers, other opinion leaders) may make it socially unprofitable for women to report violence to the necessary legal agencies that may compel the perpetrators to be accountable for their violence [4, 17, 18]. Men may be encouraged to conform to dominant masculine credentials, especially being aggressive and violent as defining traits of hegemonic masculinity. Women, by contrast, may be discouraged from reporting their violent experiences on the ground that such report has the tendency to jeopardize the reputation and social standing of their husband’s family [17].

In view of the ongoing discussion, it is essential to address the violence of men against women by foregrounding the intersection of individual attitudes, sociocultural norms, and the broader political context in which violence becomes normalized. This is an important step in developing Southern scholarship to nuance and complement global understanding of violence and to explore the possibility of mitigating the violence of men. Situated within a decolonial African feminist lens as advanced by [19], such an approach takes seriously complex and intersecting inequalities, unequal power relations, gendered subjectivities, and other contextual specificities that may contribute to the acceptance of violence. This route to knowledge production, as this article would demonstrate, has the potential to sharpen critical understandings of intimate partner violence beyond dominant Euro-American narratives. In a non-European context such as Ghana, cultural gatekeepers such as chiefs, traditional leaders and religious leaders at the local level play significant roles in providing first-line support and advice on how potential or real cases of intimate partner violence should be resolved in ways that do not disturb normative gender and family arrangements. Even as community leaders, including the *Tengdaaba* (male traditional leaders who are normatively charged with land and ritual matters in northern Ghana), chief and elders, heads of clans and family, continue to play significantly diverse roles in addressing intimate partner violence, there is very limited evidence on what exactly their roles have been, what they are currently doing to address violence, their strengths, and potential areas for improvement. In this vein, the current study is the first of its kind to explore and document in depth the roles of community leaders as key stakeholders in addressing IPV particularly in a patriarchal context with limited support systems. Findings from this study highlight important insights on how local structures can be strengthened to adequately respond to intimate partner violence.

## The socio-political and historical context of the current study

Ghana (formerly known as Gold Coast) gained independence in 1957 after enduring several decades of violent colonial rule. Ghana can be described as a predominantly patriarchal society. The country has signed and/or ratified almost all international and regional human rights conventions such as the United Nations Convention on the Elimination of all Forms of Discrimination Against Women (CEDAW) and the Maputo Protocol. Although the protection of men and women through national policies has improved over the years, gender inequalities continue to persist. For example, various feminist coalitions have been influential in lobbying the government of Ghana to pass the Domestic Violence Act (DVA). In 2007, the Domestic Violence Bill was passed into law (Act 732) after the Bill had endured in Parliament since 2003. The Act criminalizes any form of violence and persons found guilty of exacting or facilitating the perpetration of any form of gender-based violence is punishable by law. Despite what seems to be progress on the legal front, scholars [20] have noted that there is poor political commitment on the part of the Ghanaian government in sustaining the Domestic Violence Fund. The Domestic Violence Fund is supposed to support survivors of violence in seeking redress to violence. Yet, it is saddled with bureaucratic complications and delays. In view of the frustrations that survivors of violence go through in an attempt to get redress in a context where much of the population lives in rural areas with limited support services, most survivors prefer seeking help from friends, family members, and community leaders or not at all.

Notwithstanding the development in relation to legal initiatives and strategies aim at addressing violence, research has showed that gender-based violence in Ghana persists. For example, an earlier nationwide study conducted by [21] revealed that about 33% of women in Ghana have experienced multiple forms of violence perpetrated by their current or former partners. Furthermore, analysis from the Ghana Demographic Health Survey [GDHS] suggests that about 36.6% of women of reproductive age (15–49 years) reported being physically violated in the 12 months preceding the survey [22]. In Ghana, women’s vulnerability to violence is complicated by cultural norms which afford men dominance, power, and authority [4]. Additionally, women may become vulnerable to violence due to their relative lack of economic resources. Women’s lack of economic resources, coupled with cultural ideologies and heteronormative arrangements place them in relatively subordinate positions compared to their male counterparts. It is within this context that this study attempts to explore and analyze the perceptions, attitudes, responses, and reactions of community leaders towards addressing IPV.

### Research setting

Ghana is divided into sixteen administrative regions. Each region has a number of decentralized units called districts. The current study focuses on the Lambusie-Karni District of the Upper West Region of Ghana. The region has a population of about 702,110, with 51.4% being women and 48.6% being men [23]. The Upper West Region is one of the poorest in Ghana [24–26]. For instance, according to [25], the region recorded the highest incidence of extreme poverty of 45.2% which is above the national rate of 8.2%. Furthermore, the majority of the population of the region lives in rural areas where access to basic social amenities such as good roads, potable water, and electricity is limited. Rainfed subsistence farming is the main source of livelihood for the people of the Upper West Region, even though the trend of formal employment is fast becoming popular, especially in the urban areas. While other religions are practiced in Northwestern Ghana (e.g., traditional African religion, Islam, etc.), the region remains strongly a Christian society. In addition to the overwhelming embrace of Christian doctrines, values, and ideals, Northwestern Ghana in general and the Lambusie-Karni District in particular could be described as patriarchal and heteronormative [4]. Extended family system is practiced, albeit the trend of nuclear family is becoming widespread in contemporary times. Older members of the extended family, usually men have an important role in making major decisions that affect members of the family, including marriage and divorce. In view of this, although divorce is not customarily prohibited, it is strongly discouraged. The social norms governing marital relations cast divorce as less profitable and shameful. In particular, women are discouraged by societal norms and practices which are embedded in various socialization processes from seeking divorce as this leads to questions regarding their femininity and also brings ignominy to their natal families. Heads of households, chiefs, traditional and religious leaders are instrumental in endorsing or challenging patriarchal attitudes, beliefs, and behaviors that are normatively expected of men and women. These cultural gatekeepers occupy respected and influential positions in the Ghanaian society. Their opinions are usually perceived to be well-thought-out hence community members often turn to them for advice, especially on a taboo issue such as divorce.

Informed by patriarchal ideologies and heteronormative arrangements, men wield greater power and authority than women. Traditional notions of gender dictate that men are positioned as the heads and breadwinners of their families while women are constructed as subordinate subjects. Due to these perceptions, husbands are often viewed as being entitled to exercise power and control over wives in heteronormative relationships [4]. These notions of gender shape what both men and women perceive to be appropriate behaviors and traits that men and women should embody. From existing studies in the same context [4, 27, 28], evidence suggests that men are morally obliged to use violence against women when the latter fail to adhere to established gender structures, heteronormative scripts and ideals. In the event that a woman fails to abide by dominant gender scripts, the violence of men is often constructed as being “corrective” and “normal”. Community leaders are mostly men and they have a lot of authority and power in resolving cases of violence at the local level. In view of this, it is important to explore the extent to which community leaders may impose or challenge dominant cultural norms, ideologies, and values that may normalize violence in their everyday work.

## Materials and methods

This study draws on the theoretical resources of a decolonial African feminist lens to foreground how community leaders in Northwestern Ghana interpret men’s violence against women and the processes of addressing such violence. Decolonial African feminist perspectives allow us to shed light on how the intersections of history, socio-cultural norms, power inequalities, and heteronormative arrangements shape people’s meaning-making on everyday gendered subjectivities. In view of this, a qualitative research methodology was adopted as the most appropriate approach to explore and foreground community leaders’ responses, reactions, perceptions, and attitudes toward IPV. Qualitative research methodology enables the researcher to gain rich and contextually-nuanced insights on how people may interpret and make sense of their lived experiences and everyday subjectivities.

### Study procedure and participants

In order to gain rich data for analysis, the study employed a purposive sampling technique to select and recruit community leaders. This approach was deployed because the study targeted people who were considered to be at the forefront of resolving cases of partner violence at the local community level. From earlier interactions that the first author had with community members during fieldwork for a doctoral research project on constructions of masculinity and gender-based violence in December 2015, evidence revealed that community leaders, especially local chiefs play enormous roles in resolving possible cases of IPV in their respective communities. The first author took particular interest in understanding further community leaders’ own perspectives and responses to partner violence. Therefore, the inclusion criterion for this study was that all participants had to be identified as community leaders (regardless of gender, educational qualification, marital status, and age), although in this patrilineal setting all of them were men.

### Data collection

With the permission and support of the chiefs, interview dates and times were arranged. Chiefs in their respective communities agreed to mobilize members for the interviews. Thirty participants who self-identified as males agreed to participate in the study. The participants were selected from five rural communities across the Lambusie-Karni District of the Upper West Region. Recruiting participants from different communities enabled the researcher to capture diverse views and experiences.

In each community, one Focus Group Discussion (FGD) with six discussants each was held. In all, a total of five FGDs were successfully conducted with a total of 30 male community leaders. The age range of participants was between 45 and 60 years. Most participants attained a minimum of high school education and were mostly subsistence farmers. FGDs provided an important opportunity to capture diverse perceptions, beliefs, and attitudes of community leaders on partner violence. FGDs as a methodological approach also allowed the researcher to understand how community leaders may construct and make sense of violence as it is linked to dominant cultural norms and practices in their respective communities and larger society. In each FGD, specific questions on societal expectations relating to what it may mean to be a husband or wife were asked. Following this, participants were asked: (i) In your community, what amounts to intimate partner violence?, (ii) Have you ever imagined such violence to be justified under any circumstance?, (iii) What are your views on men who abuse their wives?, (iv) Do you think that the enactment of violence among men is a demonstration of masculinity?, (v) How do you resolve possible or real cases of violence in the community? In exploring these questions, the authors were interested in understanding community leaders’ knowledge of violence, their attitudes and reactions to such violence, and their responses when a potential case of violence has been reported. The researcher further engaged participants to understand the challenges and opportunities available for redressing violence. As a result of gender dynamics, all the FGDs with community leaders were conducted by the first author (a male and native speaker of Dagaare). Each FGD lasted an average of 45 minutes.

### Data analysis

All FGDs were conducted and audio-recorded in Dagaare (local language of participants), and subsequently translated verbatim into English by the first author. The second author, a female researcher with considerable experience in qualitative interviewing around sensitive topics independently transcribed the audio-recordings. To enhance the validity of the transcripts, various debriefing workshops were held with the participants after the interviews were transcribed. During the workshops, the transcripts were read to participants in Dagaare. Even as we acknowledge our positionality as members of this cultural group, we are aware of the inherent difficulty in translating words from Dagaare into English without compromising depth and richness. The feedback from the validation workshops were helpful in further clarifying some of the findings that appeared ambiguous initially.

We were wary of ongoing debates that challenge the bifurcation of inductive versus deductive approaches to interpreting qualitative data. Much like scholars such as [29], we recognize that using both inductive and deductive approaches is helpful when working with qualitative data. A hybrid approach enables us to give voice to our research participants while simultaneously ensuring that our data interact with the existing theoretical literature. In using a deductive approach, a pre-defined list of codes was created before we coded our data inductively [30]. It is important to read this process as an iterative process involving navigation between participants’ narratives and theoretically relevant literature. A blend of these approaches enabled us to accommodate new and interesting codes that emerged through the inductive coding process. Even as we aim to achieve empirical sophistication by ensuring that our codes represent the entire data, a deductive approach allows the data to stay attuned to existing theories thus enabling us to achieve theoretical triangulation [30]. In view of this epistemological rationale, we adopted the Framework Method as advanced by [31]. This framework has gained prominence in recent years as a suitable lens in making sense of textual data. The framework combines the strengths of inductive and deductive analyses while minimizing their respective weaknesses. Through this framework, we are able to compare and contrast multiple views as articulated by different participants, while also situating each view within the specific context in which such perspectives emerged.

Combining the strengths of inductive and deductive approaches, the researchers adopted and followed a six step-analytic framework as proposed by [32]. Following this analytic framework, all the authors carefully and repeatedly read all the transcripts to familiarize themselves with the diverse narratives of the participants. Based on the issues that emerged from the interviews, various codes were generated. These codes were categorized into meaningful themes and sub-themes with the aim of harmonizing and developing synergies between and among themes. After coding the data, the transcripts were analyzed using a thematic analytical framework.

Even though this study has provided critical insights into IPV from the perspectives of community leaders, it is important to recognize that the findings are only limited to a small group of leaders. Their views do not represent the views of all other community leaders in northwestern, and Ghana, more broadly. Also, the findings are limited given that relevant state agencies involved in addressing IPV, such as the Domestic Violence and Victims Support Unit of the Ghana Police Service were not sampled for the current study. Their involvement could have added a different perspective and thus enriched the findings.

### Ethical consideration

Findings from this paper form part of a larger qualitative study. Ethical approval for the study was obtained from the African Gender Institute, University of Cape Town, South Africa, where the first author received his doctoral training and degree. The purpose of the study was explained to all participants in Dagaare. Participants who could read and write in English were asked to sign a written consent form while those who could not read and write verbally agreed to participate in the study in the presence of other participants and a research assistant. In order to maintain the confidentiality of any information provided by participants during the FGDs, pseudonyms have been used throughout this paper. Before the start of each FGD, participants were also reminded that they may withdraw from the study at any time without sanction.

## Results and discussion

Dominant themes that emerged from our analyses focused on participants’ perceptions of violence, causes of violence, normative role of husbands, patriarchal norms and how such norms may justify violence, and ways of responding to violence in everyday activities. Therefore, this section is organized according to the various themes which are supported by extracts taken from the larger data. These extracts are taken verbatim to illustrate the context that may accompany specific stories and narratives relating to IPV.

### Cultural gatekeepers’ perspectives on violence against women

This sub-theme provides critical insights on community leaders’ perspectives on violence against women, especially IPV. Participants reported a range of perspectives on what may constitute IPV. Most participants thought that IPV involves the use of physical force as explained by this community leader: “What I understand about IPV is that it is when a man beats his wife. Yes, it involves physical beating like using an object to hit your wife for no apparent reason”. The views of many other participants resonate with such a perspective. When participants were asked the first issue that comes to their mind when they hear IPV, beating and hitting were immediately cited as the most common forms of IPV. Throughout the interviews, most participants suggested that an act only becomes violence when it involves the use of extreme force that has the potential to expose the victim to physical injuries. Most participants tended to conflate IPV with physical beating or using an object to hit a woman. Yet, physical violence may be an open manifestation of long-standing more subtle psychological and economic-related abuse.

Despites the views above, a few other participants articulated that IPV goes beyond just physical beating. In their reflections, IPV may involve the use of insulting words and demeaning language towards women. This was captured by a pastor:

“My understanding is that IPV isn’t just about hitting or beating your wife. Not at all. When a man uses inappropriate words to make his wife feel unhappy, that is violence. Most men are guilty of this behavior. The little mistake that your wife makes, you tell her that she is stupid. This makes her feel sad. Making someone to feel bad is violence. It is not in line with the wisdom of God”.

Another participant suggested that passing lewd comments constitutes IPV:

“I think that when a man shouts at his wife, that is not good. For example, you are with your wife in the public and you use words to make her look bad, that has a lot of emotional implication. You know, some women feel terribly hurt when you shout at them or call them names like describing her as a ‘good for nothing person’, especially in the public. When you describe her this way, it is a form of IPV because it makes the woman feel unhappy about it”.

For other participants, restricting the movement of women or forcing women to do something against their wish constitutes IPV. This was explained by this participant:

“To me, when a man forces his wife to do something against her wish, that is violence like forcing her to have sex with you when she is not ready or prepared for it. Or maybe, I am not too sure about this; when a woman wants to visit her parents and the man tells her that she cannot. I think this will make her feel bad and it is a form of violence”.

### My role as a servant of God is not to separate families, but to unite them

A major theme that was commonly articulated among participants was the place of marriage in societal meaning-making. The values that society places on marriage determine how community leaders are likely to respond to IPV within the local communities. In most cases, participants suggested that their roles in society should include counselling and advising couples to commit to each other. Drawing on dominant scriptural interpretations, a priest explained further:

Husbands and wives need to respect each other as illustrated in the Bible. The man has to be responsible and the woman also needs to be a good wife. During church service, I always make this clear: husbands and wives do not need to see themselves as competing for power or something. When women report to me that their husbands have abused them, I always encourage them to be strong in faith. My role as a servant of God is not to separate families, but to unite them as God desires. If your husband is abusive to you, that is your cross. You need to carry it. Sometimes, God wants to test our patience.

The excerpt above highlights that women who engage with some of the community leaders in patriarchal settings may be predisposed to violence. However, the quote also reverberates with discourses that draw attention to the role of Judeo-Christianity in reinforcing partner violence against women [28]. Community and religious leaders often act as important and trusted people in dealing with cases of partner violence. For example, most participants revealed that victims of violence (mostly women) would first contact them for help or advice. Community leaders were widely seen as well-respected people whose wisdom and counsel are held in high esteem. In the absence of formal referral services and shelters for victims of domestic violence, community leaders were perceived as the first point of call in the event of violence. Participants spoke extensively about how they encouraged couples to treat each other with respect and to see each other as equal partners. According to participants, encouraging couples to see each other as partners is a useful strategy in addressing marital conflict and by extension violence since cultural norms position men as the heads of households and women as subservient.

An important leitmotif across the interviews was the perception that most men are failing to live up to their cultural mandate as breadwinners. This failure is occasioned by crop failures due to erratic rainfall pattern in the semi-arid setting of the study. Yet, failing to embody responsible masculine traits, including being a breadwinner was perceived to be a main trigger of intimate partner violence as explained by one of the traditional leaders:

In our culture, men marry women. Once a man marries a woman, he is expected to take care of her. If the man fails to perform his role as the breadwinner, the woman bears the burden of taking care of the family. This can lead to conflict. If the conflict is not managed appropriately, it can escalate into violence. I have been telling the men in this community that it is their responsibility to provide for their family. If you are responsible, there will be minimal conflict. You know, violence destroys relationships.

The excerpt above suggests that a man’s inability to shoulder his responsibility as an economic provider and breadwinner appears to trigger IPV. Within this cultural context, men are normatively expected to marry women and take care of them by providing food, shelter, and other needs. By this problematic cultural arrangement, men are often framed as powerful and in control of their families. Within the context of this study, where dominant milestones required for men to ascend to the position of economic providers and breadwinners is fast declining, there is strong possibility that men are likely to feel inadequate and disempowered when they fail to measure up to such societal standards. In such a situation, men may consider deploying acts of violence to reassert their dominant and powerful position as well as stabilize the boundaries. Informed by structural inequalities and cultural perceptions on masculinities and femininities, women who ‘usurp’ the position of their husbands as the breadwinners are likely to create a reversal of gendered power dynamics and conflict. This is so because men are traditionally socialized to serve as breadwinners and not to be economically taken care of by women. To be taken care of by a woman as a man may be read as emasculating, considering the local norms governing masculinity and femininity.

### ‘There is no smoke without fire’: Reproducing problematic heteronormative discourses

This study found out that women were often advised by community leaders whose perspectives hold sway in their lives to believe in themselves and imagine a better future for themselves and their children. According to the participants, women should have the courage to sustain their marriages irrespective of the predicaments. Throughout the interviews, groundnut/peanut was the metaphor to which participants frequently likened marriage. From the outlook of groundnut/peanut, one is unable to predict whether the seeds inside are good or bad. Once it is opened, one needs to accept it without questioning. Translating this into marriage, participants suggested that once a man and woman accept to marry each other as husband and wife, they should be ready to accommodate both the good and bad outcomes of their choices. While most participants thought that men have an important role to play in minimizing violence, most of them tended to focus on advising women on what to do to make the marriage succeed. Sustaining a marriage was constructed as the responsibility of the woman. Informed by such perceptions, most participants believed that women often provoke their husbands to become violent because it is believed that women often fail to meet marital expectations. This was explained by a priest:

God created a woman and man for good reasons; to love and to cherish each other. This means that there are times the man has to lower himself for the marriage to work. The woman too has to lower herself by ensuring that she is patient and submissive to her husband. As a father to everyone in the church, I always advise couples to truly commit to their relationship. This is important in building peaceful and non-violent families. If your husband is abusive to you, you would need to examine yourself as a woman. You would need to reflect on this and have a change of behavior.

Another participant, a traditional ruler, agrees with the narrative of the priest above, adding that:

When cases of violence are brought to my notice, I always tell the couples that: ‘two wise people cannot stay under the same roof’. One has to be a fool in order to make the marriage work. If both of you think you’re always right; then, there is a problem. There is higher risk of violence to occur because no one wants to be seen as a loser. In a marriage, there is no loser or winner. As a community leader, I see it as my responsibility to encourage husbands and wives who have disagreements to sit down, dialogue, and look into the problem. Once you identify the cause of the conflict, both of you need to work on resolving it together.

Another chief from a different settlement explained further:

We cannot run away from violence. What we can do, in my view, is to minimize it. While I think that both men and women have roles to play in minimizing violence, women have a lot to do. When a woman shows good attitude and behavior to her husband, I don’t think her husband can just get up and start beating her. Absolutely not! However, when a woman shows an unacceptable behavior, she must be corrected to avoid future occurrence.

The perception that women always provoke their husbands to be violent was widely articulated in other interviews. Most participants expressed the view that violence does not happen for no apparent reason. This is demonstrated in the narrative of another chief:

‘There is no smoke without fire’. When couples bring their issue to me, I always ask the woman “What did you do for your husband to beat you?” You see, men have their ego. As the head of the household, no man wants to be looked down upon by his wife. So, if she says something that disturbs his ego, it could trigger violence. In that case, I always advise her not to repeat that bad behavior. Women who want to show their ego in the face of their husbands get beaten. It is not that I support the violence of men, but this is the reality.

Throughout the interviews, it became evident that community leaders, such as chiefs, traditional rulers, religious leaders, etc. who play diverse roles in settling cases of violence against women within their respective communities have no guidelines to inform their decisions. They largely draw on their own judgement, repertories of cultural values, and experience in resolving IPV in ways that do not contribute to the separation of families.

Without involving relevant state apparatuses, such as the Ghana Police Service in resolving cases of violence, there is significant risk of reproducing power hierarchies and gendered norms, which disadvantage women as demonstrated by the comments above. From the narratives presented above, community leaders fail to provide any concrete responses to victims of violence, especially women. There is clear evidence from this study to suggest that community leaders often fail to actively listen to the lived experiences of women in their judgements. Failing to take seriously the lived experiences of women in their judgement reproduces a victim blaming discourse (“if she says something that disturbs his ego, it could trigger violence”). Such victim blaming discourse was strongly illustrated when participants suggested that women needed to be “patient” and “submissive” to their husband. Women needed to demonstrate that they are “good wives” as imagined in the Bible and Dagaaba cultural norms in order to minimize violence against them. Women who fail to embody traditionally “feminine” credentials were perceived to be at greater risk of experiencing violence as illustrated by the view: “Women who want to show their ego in the face of their husbands get beaten”.

The narratives of participants as articulated above should be read as situated attempts in their position as community and religious leaders to balance their supposed rejection of men’s violence against women. Participants appeared to be in a dilemma, where they attempted to negotiate two contradictory positionalities. Participants struggle to reconcile and make sense of what seems to be conflict between their call to condemn violence and men’s privileged positions within a gender inequitable society. Informed by their own access to male power, privilege, and authority, the effectiveness of participants’ calls to reject violence become ultimately limited. Masculine privilege thus becomes socially constructed and enacted over time within durable patterns and hierarchies of relationships. Being aware and critical of masculine privilege as an important ingredient of an unequal gender order is a critical approach in rethinking the complexity and dynamics of violence as [16] has pointed out. Violence, as a product of masculine privilege, is enacted at multiple levels through either acts of violence, nonviolence, or both. In view of this, researchers interested in gaining a deeper understanding of partner violence should foreground acts of violence as culturally, interpersonally, and institutionally sanctioned.

### “Those who cause the problem must solve the problem together”

Most participants alluded that relying on the local structures appears to be the most effective way of resolving partner violence and minimizing divorce. Across all the sampled communities, participants claimed that violence has reduced because most men are beginning to see the benefits of healthy, peaceful, and nonviolent relationships. This was explained by a chief:

In every marriage, there is bound to be disagreements. If such disagreements are not managed well, violence may occur. In my view, when there are disagreements, the couple needs to listen to and be patient with each other. Why is it that did not go down well with my partner? As a community leader, I am a father for all. I always advise them to respect and commit to each other. This is very important in sustaining marriages. Most men are beginning to see the importance of this. They now see that violence is not good. The cost of violence is more than its benefits.

Similarly, other participants thought that the advice and moral encouragement from community leaders was an effective approach in keeping couples together and without violence. Community leaders were perceived to be crucial figures in helping to settle couples’ differences and foster healthy relationships. In their reflections, it was important for disputing couples to develop and practice forgiveness. In demonstrating why it was necessary for couples to develop and practice the spirt of forgiveness, a religious leader narrated that: “Those who cause the problem must solve the problems together”. Most community leaders believed that this expectation of the marital couples to work towards settling any differences that could potentially escalate into violence was a necessary strategy for keeping family issues secret. In their estimation, reporting cases of violence to the police is likely to unsettle marital unions in the family and broader community. Participants spoke about how they fear that relying on the formal legal approach in resolving violence is likely to be less helpful in uniting families as narrated by one village chief:

If we report the couple to the police and the man is fined or imprisoned, the marriage may break down. The man may not want to accept the woman back as his wife again. There will be bad feelings between the man and the woman’s families. If the man is imprisoned, who will take care of the children? The woman cannot take care of the children all by herself. As community leaders, this is not what we always aim for; we want the couple to realize their own mistakes, make amends, and live happily.

Most participants perceived that the formal approach tends to focus on punishment and separation of families in accordance with the laws of Ghana. In their reflections, couples who have disagreements are supposed to find solutions to their own problems by themselves rather than involving the police and other external people. In encouraging couples to draw on local structures to find solutions to their disagreements without resorting to the police, participants suggested that couples need to dialogue, listen to each other, and admit their wrongs when necessary. This dialogue was perceived to be an important strategy in minimizing violence. Consider an explanation given by one of the village chiefs:

We want couples to recognize their mistakes. As a community leader, my interest is to guide couples on how to resolve their disagreements by themselves without necessarily getting outsiders involved. Most people believe that the police always aim at punishment and divorce. Sometimes, when we realize that it is the man who causes the disagreement, but is failing to admit it, we tell him in the face: ‘you’re the cause of the problem’. We counsel him to desist from the violent behavior. We want him to change his behavior and become a committed husband. This is not the case when the police are involved.

Throughout the interviews, participants suggested that rural patriarchal norms and the notion of family as a private entity constrain couples’ readiness to report disagreements to local authorities. Most participants expressed the feeling that violence is not a new phenomenon; it has existed in families for years as highlighted by the narrative of a traditional ruler:

Everyone knows that violence happens in every marriage. The issue is that people never want to open their problems to outsiders. It must be handled well because people’s and families’ reputations are at risk. When you involve the police, you are inviting other people to know about the secret of your marriage.

In the context of this study, marriage arrangements are patrilocal, meaning women marry into and live with their husband’s natal family. Informed by local notion of family privacy, participants explained that involving the police in cases of violence risks making the violence of men an open secret. The overwhelming embrace of Christian doctrines and ideals as well as pervasive cultural norms that valorize marriage mean that separation and divorce are discouraged in the context of this study. It was widely believed that women should be resilient enough to withstand the challenges that may attend marriages no matter the situation. Such perceptions have emerged to be central to local constructions of respectable femininity. In a context in which, at least in theory, most women depend on their husbands for economic and material needs, it will be a precarious decision for a woman to seek divorce since doing so would further increase her burden.

## Conclusions

This study has attempted to nuance the interplay of the Dagaaba people’s constructions of gender, patriarchies, culture, and intimate partner violence in the context of northwestern Ghana. The findings in this study demonstrate that community leaders (chiefs, traditional leaders, religious leaders) as critical cultural gatekeepers are knowledgeable about the problem of intimate partner violence in their respective communities. Based on their own perceptions of such violence as a common phenomenon, the findings highlight the diverse and complex ways in which community leaders are likely to challenge or sanction intimate partner violence in their everyday activities. In articulating their reactions, attitudes, and responses to violence, participants pointed to complex scenarios in which the possibility to explore and make use of relevant legal actions against violence perpetrators is likely to be compromised. While all participants appear to condemn men’s violence against women as unacceptable, their readiness to challenge such violence was potentially compromised because none of them wanted to be stigmatized as being responsible for marital breakdown or broken families in a setting where marriage is priced. Messages of ambivalence in responding to violence emerged throughout the interviews. Such perspectives of ambivalence profoundly shape how community leaders are likely to advocate for the legal rights and support for victims of violence at the individual and community level. In order to minimize the risk of being tagged as advocate for the marital breakdown, most community leaders are likely to reinforce patriarchal ideologies and traditional gender norms and attitudes which tend to normalize partner violence against women through the use of customary dispute resolution mechanisms. Unfortunately, customary dispute resolution mechanisms in the context of this study appear to be unfavorable to women as victims of violence. By leveraging on such informal mechanisms, the findings in this study highlight the multiple ways in which community leaders may individually or collectively reproduce dominant cultural tropes that normalize violence. Despite the cultural and geographic variations, our findings are consistent with existing literature which attributes the occurrence of men’s violence to a range of interlocking factors, including entrenched cultural beliefs, patriarchy, community norms, and women’s dependence on men for material needs [2, 4, 6, 33, 34].

Our findings unpack how structures of patriarchy work in tandem with dominant constructions of masculinity and femininity to shape community leaders’ politics and responses to violence. Our findings offer useful insights in understanding how gender hierarchies and the notion of culture as embodied in this part of Ghana may have significant implications for domestic violence policy and interventions.

The article concludes that attempts to address intimate partner violence in a patriarchal context such as Ghana must first focus on disrupting and challenging patriarchal cultural norms that make violence flourish. Policy needs to be localized and refocus the gaze in changing social norms. As argued by [33, 34], such an approach requires a critically sympathetic understanding of how individuals perceive certain norms and practices in ways that may promote social change and liberatory discourses. Using proactive measures that do not dismiss local structures, this can be done through sustained and active education at the community level. People should be educated on the importance of promoting peaceful and non-violent masculinities.

While our findings are not generalizable to the larger Ghanaian society, an important message that should be taken seriously in gender-based interventions in a patrilineal northwestern Ghana is being sensitive to local cultural norms, practices, and heteronormative arrangements. Based on such evidence, we would argue that if policy makers, development practitioners, and researchers are to adequately and sustainably address the violence of men, a useful starting point is to build on community leaders’ perspectives, attitudes, and responses to violence. By building on their perspectives, responses, and reactions in their everyday work, we may be able to challenge and deconstruct the multiple ways in which community leaders’ approaches to addressing violence are reinforcing gendered subjectivities and experiences. It is imperative for community level education and training to sensitize community leaders about the harmful consequences of violence and the need to put in place structures that will enable victims of such violence to report to the necessary state legal apparatus without dire repercussions. Despite the grave consequences that may attend such reporting of violent male partners, this proposal is achievable in the long-run with sustained edification.
